# A Comprehensive Review of Cancer MicroRNA Therapeutic Delivery Strategies

**DOI:** 10.3390/cancers12071852

**Published:** 2020-07-09

**Authors:** Alexis Forterre, Hiroaki Komuro, Shakhlo Aminova, Masako Harada

**Affiliations:** 1 UMR DIATHEC, EA 7294, Centre Européen d’Etude du Diabète, 67200 Strasbourg, France; a.forterre@ceed-diabete.org; 2 Department of Cardiovascular Physiology, Tokyo Medical and Dental University, Tokyo 113-8510, Japan; hkomcvp@tmd.ac.jp; 3 Lyman Briggs College, Michigan State University, East Lansing, MI 48825, USA; aminovas@msu.edu; 4 Institute for Quantitative Health Sciences and Engineering (IQ), Michigan State University, East Lansing, MI 48824, USA; 5 Department of Biomedical Engineering, Michigan State University, East Lansing, MI 48824, USA

**Keywords:** microRNA delivery, cancer therapy, therapeutic RNA, extracellular vesicles

## Abstract

In the field of molecular oncology, microRNAs (miRNAs) and their role in regulating physiological processes and cancer pathogenesis have been a revolutionary discovery over the last decade. It is now considered that miRNA dysregulation influences critical molecular pathways involved in tumor progression, invasion, angiogenesis and metastasis in a wide range of cancer types. Hence, altering miRNA levels in cancer cells has promising potential as a therapeutic intervention, which is discussed in many other articles in this Special Issue. Some of the most significant hurdles in therapeutic miRNA usage are the stability and the delivery system. In this review, we cover a comprehensive update on the challenges and strategies for the development of therapeutic miRNA delivery systems that includes virus-based delivery, non-viral delivery (artificial lipid-based vesicles, polymer-based or chemical structures), and recently emerged extracellular vesicle (EV)-based delivery systems.

## 1. MicroRNA Overview

The central dogma of molecular biology was no longer “central” after the discovery that gene expression involves more complex layers of regulation than the DNA sequences. MicroRNAs (miRNAs) are one of the most well-studied non-coding RNAs that play a critical role in gene regulation, some of which are dysregulated in association with certain cancer types. The first cancer-associated miRNA was discovered from the study on the commonly deleted region 13q14 in B-cell chronic lymphocytic leukemia (B-CLL), where the minimally deleted region among patients located at first exons of two non-protein coding RNA transcripts, DLEU 1 and DLEU 2 (Deleted In Lymphocytic Leukemia 1 and 2), aligned in opposite directions [1]. In 2002, Calin and his colleagues identified two miRNAs, miR-15a and miR-16-1, located within the intron of the DLEU2 gene that was deleted or downregulated in the majority of CLL patients [2]. Further studies revealed that these miRNAs function as tumor suppressors to induce apoptosis by repressing the B-cell lymphoma 2 (Bcl-2) gene, and inhibit the cell cycle by the repression of cyclin-related genes such as CCND1 and CCNE1 [3,4]. These discoveries sparked the field of miRNA-cancer research, the exploration of a unique miRNA transcriptome for each cancer type, empowering the better classification of specific cancer types and prognostic information.

The canonical pathway of miRNA biogenesis initiates from a capped and polyadenylated long primary transcript (pri-miRNA or pri-miR) transcribed by RNA polymerase II (Pol II), resembling that of protein-coding transcripts [5]. A stem-loop structure of pri-miRNA is recognized and further processed by the nuclear RNaseIII enzyme—a double-stranded RNA binding domain protein (DROSHA-DGCR8) complex—into a hairpin-loop structure, called precursor-miRNA (pre-miRNA or pre-miR) [5,6]. Pre-miR is exported from the nucleus by the Exportin-5-Ran-GTP axis and further processed into the mature form of double-stranded RNA by Dicer-TRBP complex [7]. RNA-induced silencing complex (RISC) containing Argonaute (Ago) proteins incorporates one strand of the miRNA duplex and guides it to the partially complementary site within 3’UTR of the target gene [8]. There are several non-canonical pathways of miRNA biogenesis including a miRNA derived from an intronic region of mRNA by splicing (Miltron) independent of Drosha processing, and a miRNA trimmed by poly(A)-specific ribonuclease (PARN) independent of Dicer cleavage [9,10]. Nevertheless, the vast majority of miRNAs fall into the former category of biogenesis, in which processes and machinery are highly conserved across evolutionarily distant species.

There were reports on correlations between the dysregulation of miRNA machinery and disease progression and poor prognosis in several tumor types such as ovarian cancer [11], neuroblastoma [12] and lung cancer [13]. In most cases, cancer cells exhibit a distinct miRNA pattern that contributes to specific traits of malignancies, possibly because other RNA species share the same regulatory pathway. MiRNA binding through a partial complementary sequence (seed sequence) within the target mRNA 3’UTR primarily inhibits translation or promotes its degradation. A specific miRNA can regulate hundreds of genes, but in reality, only 30–40% of genes are regulated by miRNAs and the target sites are often selectively conserved within the 3’UTR [14,15]. There are a couple of reports on miRNA-mediated gene activation or miRNA binding to non-3’UTR region, which appear as infrequent cases [16].

MiRNA regulation: Genomic loci of miRNA can be either intergenic when they have their own promoter driving their expression or intragenic where the miRNA gene is within the intron or exon of a protein-coding gene. According to the integrating miRNA inter- and intragenic database (miRIAD), of 1871 human miRNAs, 1072 (57.3%) are intragenic and 799 (42.7%) are intergenic [17]. The transcriptional regulation of miRNA genes is much like that of protein-coding genes, determined by factors including transcription factors, DNA methylation, mutations, copy number variations, RNA stability and cleavages [18,19,20]. One such example is when glucocorticoid, one of the commonly used steroid hormones to treat hematopoietic malignancies, binds to its receptor, and the receptor complex binds to the upstream promoter region of the miR-17-92 cluster gene resulting in transcriptional repression [21]. On the contrary, miRNAs’ specific regulatory mechanism involves miRNA biogenesis or processing machineries, often leading to a global miRNA alteration associated with human cancers. Such dysregulation of RNaseIII type enzymes, nuclear DROSHA and cytoplasmic DICER, as well as components of the RISC complex, TARBP2 and AGO2, were reported in association with various types of cancers [22]. The emerging role of the growth-promoting tumor microenvironment involves intra-cellular miRNA regulation [23]. Recent research reported that miRNAs play key roles in the crosstalk between cancer-associated fibroblasts (CAFs) and tumor cells, which were transferred by cell-derived extracellular vesicles. The extracellular miRNAs from cancer-associated fibroblasts granted favorable traits such as growth promotion, survival and drug resistance in various tumor types, including colorectal cancer, breast cancer and pancreatic cancer [24,25,26,27]. In summary, the fine tuning of miRNA levels is comprehensively coordinated by both internal and external regulatory mechanisms [28].

## 2. Altered MicroRNA Expression in Cancer Cells

### 2.1. Altered miRNA Profile in Malignancies

Over the past decade, it has come to light that miRNAs present altered expression patterns in malignant tissues and cells. Some miRNAs regulate essential genes for cellular homeostasis in which the alteration will result in abnormal biological changes, including uncontrolled cell proliferation, angiogenesis, metabolism and apoptosis, leading to malignant formation. All of the key cancer pathways have an association with miRNA alterations; sensitivity to growth signals (e.g., let-7 family, miR-21); insensitivity to antigrowth signals (e.g., miR-17-92 cluster, miR-195); apoptosis escape (e.g., miR-34a, miR-185, miR-15/miR-16); angiogenesis (e.g., miR-210, miR-26, miR-15b, miR-155); invasion and metastases (e.g., miR-10b, miR-31, miR-200 family, miR-21, miR-15b); evading immune destruction (e.g., miR-124, miR-155, miR-17-92); tumor-promoting inflammation (e.g., miR-23b, miR-155, let-7d); and genomic instability (e.g., miR-21, miR-155, miR-15b) [29,30,31]. The malignant transformation will alter cell- and cellular state-specific miRNA expression profiles in healthy tissues. Furthermore, miRNAs are capable of driving malignant formation either by repressing tumor suppressor genes or increasing oncogene expression, thus emphasizing the importance of miRNAs in malignant transformation and as biomarkers to further subclassify cancer types [32,33,34].

Comprehensive miRNA expression analyses revealed a set of miRNAs that show distinct expression patterns in various cancer types, which led to biomarker discovery, or further functional validation of these miRNAs. For example, global miRNA expression analysis of diffuse large B-cell lymphoma (DLBCL) revealed a reduced level of miR-150 and miR-29b [35,36]. Following this finding, Xiao et al. identified that miR-150 targets transcription factor c-Myb, essential in the development of lymphocytes [37], and Li et al. showed that miR-29b regulates protein kinase B (Akt) which promotes survival and growth through the PI3K/Akt signaling pathway [38]. Therefore, comprehensive miRNA expression analysis of cancer cells holds great potential for therapeutic target and biomarker discovery.

Tumor-related miRNAs are broadly classified into two groups: Oncogenic miRNAs (OncomiRs), and Tumor Suppressor miRNAs (TS-miRNAs, TS-miRs). Most, if not all, types of cancers display an induced expression of some oncomiRs, which promote tumorigenesis by blocking the translation of tumor-suppressive mRNAs (e.g., miR-17-22, miR-125b, and miR-125) [39,40,41], whereas the reduced expression of TS-miRNAs (e.g., miR-133a, miR-145, and miR-143) directly inhibits the translation of oncoproteins [42,43]. Thus, miRNA expression versatility emphasizes their utility in cancer therapeutic strategies. Therefore, antisense-miRNA (anti-miRs) or miRNA mimics were developed for miRNA therapy and are widely used to repress oncomiRs or to restore TS-miRs, respectively [44,45,46].

MiRNAs are found in both intracellular and extracellular regions of the body. Recently, several studies have revealed the potential of circulating miRNAs as a new class of biomarkers. For example, both the tissue and sera of oral cancer patients presented induced elevated levels of miR-31, which target NAD-dependent deacetylase SIRT3 that regulates metabolism and energy production [47,48]. Both tissue and sera from gastric and germ cell cancer patients exhibit elevated miR-371-3, which promotes tumor growth and metastasis by directly targeting tumor-suppressor gene TOB1 (transducer of ERBB2, 1) [49,50]. Additionally, chemotherapy resistance-related miRNA alteration was reported, miR-155 and miR-221/miR-222 overexpression was associated with a poor outcome in lung cancer and poor therapeutic outcomes of anti-estrogenic therapies (Tamoxifen, Fulvestran), respectively [51]. The implication of miRNAs in diagnosis and therapy differs over a wide range of cancer types.

### 2.2. MiRNA Editing as Cancer Therapy

Certain miRNAs are master regulators of tumorigenesis in some cancers, and thus represent powerful therapeutic targets.

For example, supplementing the reduced miR-29b, the regulator of cell growth, proliferation, and angiogenesis, caused tumor reduction in xenograft mice of breast and gastric cancers [38,52]. MiR-34a is another therapeutic miRNA that is generally under-expressed in many cancer types, notably breast cancer. Some potent targets of miR-34a include transcription factor, Fos-related antigen (Fra-1) that regulates apoptosis/proliferation/differentiation, and NAD+ dependent histone deacetylase Sirtuin-1 (SIRT1) that deactivates tumor suppressor p53 [53,54]. A phase I clinical trial using miR-34a showed antitumor activity in a subset of patients with refractory advanced solid tumors with pre-dexamethasone treatment, which is discussed in more detail later in this review [55].

The dysregulation of the miR-15a and miR-16-1 cluster involves multiple oncogenic phenotypes such as cell survival, proliferation, and invasion by directly targeting CCND1 (cyclin D1), WNT3A, and BCL2, evident in prostate and pancreatic cancers. WNT3A is part of the Wnt/beta-catenin signaling pathway responsible for cell–cell adhesion, and the promotion of oncogenes like c-Myc and CCND1. Thus, miR-15a re-expression showed a decreased viability of pancreatic tumor cells, and its knockdown resulted in an increased invasion and proliferation in prostate cancer in vivo models [56,57].

While the role of miRNA as a regulator of various physiological processes is evident, challenges lie within its delivery. In a body, crude miRNAs are unstable due to various ribonucleases in the blood, if not cleared by phagocytosis through the reticuloendothelial system (RES). In addition, crude miRNAs are unable to cross the cell membrane or the vascular endothelium due to their negative charges. To overcome these problems, various approaches were examined to deliver miRNAs. We will provide examples of in vivo experimental approaches to modulate miRNA levels across multiple tumor models for therapeutic applications in the following sections.

### 2.3. MiRNA Inhibition Therapies for OncomiRs

Reducing oncomiRs, frequently overexpressed in human cancers, allows for the restoration of target tumor-suppressor expression that could work as a therapeutic strategy. Commonly used miRNA inhibitors, single-stranded antisense, anti-miR oligonucleotides (AMOs), locked nucleic acid (LNA) anti-miRs, antagomiRs, miRNA sponges and small molecule inhibitors of miRNAs (SMIRs), interfere with miRNA biogenesis or block miRNA-mRNA interaction [31,58,59,60,61]. For instance, the intravenous injection of antagomiRs against miR-16, miR-122, miR-192, and miR-194 significantly reduced their endogenous expression without provoking an immune response [62]. Another example is with the oncogenic miR-21 antagonist, repressing AKT and subsequent Mitogen-activated protein kinase (MAPK) pathway activation, which hampered Epithelial–mesenchymal transition (EMT) and angiogenesis in breast cancer [63,64,65]. MiRNA sponges (RNA molecules with repeated miRNA binding sequences that can sequester the miRNAs of choice) successfully repress miR-23b expression both in vitro and in vivo, reducing glioma angiogenesis, invasion, and migration [66].

### 2.4. MiRNA Replacement Therapies for Tumor-Suppressor MiRNAs

Supplementing tumor-suppressive miRNAs in cancer cells is another therapeutic approach for miRNA alteration. The use of synthetic double-stranded miRNA mimics, pre-miR, or plasmid-encoded miRNA genes compensates for lost tumor-suppressor miRNAs in order to target tumor-promoting mRNAs [31,44]. The miR-34 family, defective in about half of human cancers, plays a critical role in the suppression of tumor development [67]. The delivery of miR-34 mimics to cancer cells showed a growth inhibitory effect, and confirmed the promising use of miRNA mimics in cancer treatment [67]. However, the challenges for miRNA therapy need to be addressed. As previously mentioned, miRNAs are unstable in the body due to multiple ribonucleases and RES clearance. Moreover, their negative charges impede the crossing of the cell membrane or the vascular endothelium. Even though they reach inside of a cell, they are subject to endolysosomal degradation. To ensure effective treatment in cancer cell-specific delivery, it is crucial not to disrupt the healthy tissue [68]. Impaired blood perfusion in tumors lowers systemic delivery of miRNAs, while the tumor microenvironment acts as a barrier and disrupts efficient miRNA delivery [69,70]. Tumor-associated immune cells (macrophages, neutrophils and monocytes) in the tumor microenvironment can nonspecifically uptake miRNAs encapsulated in the delivery system [65,71]. To overcome these problems, researchers have tried different approaches in delivering therapeutic miRNA mimics or inhibitors.

In the following sections, we will provide several examples to modulate miRNA levels, using in vivo models for therapeutic applications. Table 1 shows the summary of miRNA mimics or inhibitors tested in vivo via different delivery methods.

## 3. Approaches for MiRNA Therapeutic Delivery

### 3.1. Local Delivery

Due to its bioavailability and reduced toxicity, the intratumoral injection of miRNA mimics or inhibitors are significantly more effective than those delivered by systemic routes. However, local administration is limited to the localized and readily accessible primary solid tumors such as melanoma, breast and cervical cancers. One advantage of local delivery is minimal nonspecific uptake by healthy organs, which can lead to unwanted toxicity and immunogenicity.

Halle et al. presented the anti-tumor effect of intratumoral convection-enhanced delivery (CED) of a 2′-O-methoxyethyl anti-miR-let-7a in combination with phosphodiester/phosphorothioate backbone using a highly invasive glioblastoma (GBM) orthotopic xenograft model. Importantly, no animals showed signs of severe adverse effects while retaining the functional anti-miR effect following one month of anti-miR administration [95].

Trang et al. showed that both the intratumoral and intravenous administration of let-7a mimics led to decreased tumor size in the non-small-cell lung cancer (NSCLC) mouse model [75,96]. In hepatoma xenograft models, intratumoral administration of cholesterol-conjugated 2′-O-methyl-modified miR-375 mimics suppressed tumor growth by targeting the AEG-1 gene (astrocyte elevated gene-1) [97]. The same group performed intranasal delivery of the lentiviral vector expressing let-7a using the aggressive human NSCLC xenograft model, resulting in an increased let-7 expression followed by the growth inhibition of KRAS-dependent lung tumors. Additionally, the local delivery of synthetic let-7b using a polymer-based delivery triggered a specific inhibitory response leading to tumor reduction by 60–70% [96]. The polyethylenimine (PEI)-mediated local application of miR-145 achieved a significant anti-tumor effect in colon carcinoma mouse models [79]. Lastly, Nanoparticles -mediated intratumoral delivery of siRNA specific to DCAMKL-1 induced expression of let-7a and miR-144 and subsequent repression of proto-oncogenes c-Myc and Notch-1 in colorectal cancer xenografts, thus resulting in tumor growth inhibition [98]. Local administration is limited to accessible tumors; thus, the development of a systemic delivery approach is necessary to treat other types of cancers and metastatic tumors.

### 3.2. Systemic Delivery

The ideal delivery system should protect miRNAs from early degradation in the bloodstream, carry them to the target cells, and facilitate cellular uptake without inducing any immunogenic response [99,100]. To this end, oligonucleotide editing and systemic miRNA delivery systems were explored as improved therapeutic deliveries of miRNA mimics and inhibitors. The chemical modification of miRNA oligonucleotides enhances miRNA stability and prevents their degradation by nucleases in the circulation. For example, an addition of 2′-O-methyl (2′-O Me), 2′-O-methoxyethyl or 2′-fluoro to the 2′-OH in riboses, enhances the stability and binding affinity of anti-miR, and provides long-lasting effects in the liver, lung, kidney, heart, intestine, fat, skin, bone marrow, muscle, ovaries and adrenals [101]. LNAs are another type of chemically modified RNA nucleotide analog with high nuclease resistance, applicable to siRNA and miRNA mimics/inhibitors. The systemic administration of the LNA-anti-mir-122 showed a dose-dependent downregulation of liver-specific miR-122 levels in mice liver for several weeks, and the upregulation of a set of target mRNAs [102]. LNA-anti-miR-122 prevents hepatitis C virus (HCV) replication, and is the first miRNA drug to successfully enter Phase II trials for the treatment of HCV infection. In turn, this represents a promising preventive therapy for hepatocellular carcinoma (HCC) resulting from HCV infection [103]. However, such modified miRNAs have short half-lives because of the rapid renal and hepatic clearances resulting in limited tumor uptake and biodistribution [65]. In 2016, Teplyuk et al. tested three delivery routes for the miR-10b antisense oligonucleotide inhibitors (ASO): 1) direct intratumoral injections, 2) continuous osmotic delivery and 3) systemic intravenous injections in intracranial human glioma-initiating stem-like cells (GSC)-derived xenograft and murine GL261 allograft models in athymic and immunocompetent mice, both resulting in the miR-10b target genes (i.e., MBNL1-3, SART3, and RSRC1) increased expression thus inducing the reduction of growth and progression of intracranial GBM with no significant systemic toxicity when using the ASO local or systemic administrations [104].

Viral (3.3) and non-viral (3.4) vectors are commonly used vectors for miRNA delivery [41,105]. Although viral vectors are popular due to their efficiency, the adverse effects from immune responses are inevitable. Thus, non-viral vectors are the preferred choice for clinical studies. Moreover, the formulation of miRNAs in nanoparticles is an alternative strategy investigated by many groups [106].

### 3.3. Viral Delivery

Viral vectors transfer pre-miR or mature miRNA cloned in a plasmid to the tumor cells, generate mature miRNA, drive expression with the viral promoter, and subsequently repress and/or degrade target mRNAs [41]. Lentivirus, adenovirus and adeno-associated virus (AAV)-mediated delivery systems have successfully delivered either miRNA mimics or antagonists to the nuclei of tumor cells, followed by miRNA expression and function [107]. Kasar et al. showed the lentiviral delivery of miR-15a/16 to a New Zealand Black (NZB) mouse model of chronic lymphocytic leukemia (CLL) restored expression of these miRNAs whose expression were lacking in CLL [108]. Another study showed the intravenous administration of lentivirus containing miR-494 antagonists, which reduced the activity of myeloid-derived suppressor cells (MDSCs) through miR-494 inhibition, which is pro-angiogenesis and promotes tumor growth, in a murine breast cancer model [109]. Genetic manipulation allows for the conjugation of targeting moieties to the viral capsid proteins, which improves the affinity between viral vectors and cancer-specific receptors for specific delivery into the tumors [65]. Nevertheless, one should keep in mind that lentiviruses integrate their viral DNA into the host genome, which could lead to mutagenesis and the activation of oncogenic pathways. In contrast, AAVs are an attractive alternative for therapeutic delivery because of their nature, keeping their genomes in an episomal form. Kota et al. showed that the AAV-mediated delivery of miR-26a, known for its tumor-suppressing property, in an HCC mouse model resulted in the significant reduction of tumor growth without toxicity [68]. Although viral vectors can efficiently deliver functional miRNA antagonists or miRNA mimics into tumor tissues, the immunogenic response remains a serious concern in clinical applications. Thus, various non-viral delivery methods were developed as a safer alternative for miRNA delivery [110].

### 3.4. Non-Viral Delivery

Compared to viral delivery, non-viral methods are generally inefficient in miRNA transfer with an even shorter efficacy in target gene repression. However, recent advancements in the field have improved miRNA delivery by chemical modifications, making them suitable for clinical applications [65]. Non-viral vectors are classified broadly into these groups: polymeric vectors (PEIs, polylactic-co-glycolic acid/PLGA, chitosans and dendrimers) [111], lipid-based carriers (neutral/cationic and targeting-modified), biomaterials [112], and inorganic materials (gold, diamond, silica, and ferric oxide) [113].

### 3.5. Nanoparticles

Among non-viral vectors, nanoparticles are the most common choice in delivering exogenous nucleic acids, including DNA, mRNA, siRNA and miRNA, as seen in recent studies [114,115]. Despite their popularity, technical problems, including safety and efficacy, restrict its clinical usage as a therapeutic vector [116]. Nanoparticle-mediated miRNA delivery must overcome hurdles in the body, such as degradation, rapid renal clearance, endosomal escape, and pharmacokinetics for efficient delivery [110,117,118]. In particular, nanoparticles accumulate in tumors through the enhanced permeability and retention (EPR) effect, where the enriched targeting highly depends on the size of nanoparticles [119]. When designing nanoparticles, these problems need to be addressed. The following section will introduce some of the improved nanoparticle delivery systems.

#### 3.5.1. Lipid-Based Vectors

**Figure 1:**
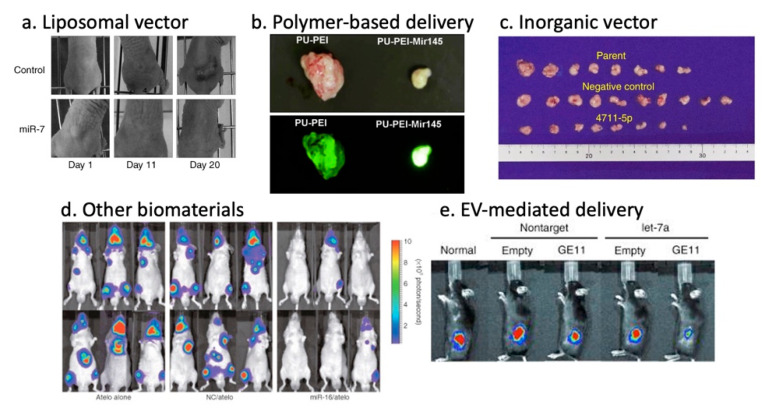
Potent anti-tumor effects of miRNA therapy through various delivery methods. (**a**) Local liposome-miR-7-plasmid treatment of xenograft mice [73]. (**b**) In vivo tumorigenicity assessment of polyurethane-short branch polyethylenimine (PU-PEI)-mediated miR-145 delivery to patient-derived Glioblastoma (GMB) cells [78]. (**c**) Tumor sizes of xenograft mice after systemic super carbonate apatite (sCA)-mediated miR-4711-5p delivery [84]. (**d**) Inhibition of metastatic tumor growth in bone tissues by the atelocollagen-mediated miRNA treatment [93]. (**e**) Inhibition of breast cancer development in vivo using GE11-positive exosomes containing let-7a [91].

Lipid-based vectors are the most widely used nanoparticle for in vivo gene therapy to date, which form spherical structures composed of phospholipid bilayers with an aqueous core encapsulating nucleic acids-drugs [120]. The advantages of liposome-based nanoparticles include biocompatibility, flexibility, low immunogenicity, and versatility of administration routes. The efficiency depends on physicochemical properties such as particle size, surface charge and lipid composition. Recent advances in the understanding and modeling of liposomes have enabled adjustments of their performance parameters, such as pharmacokinetics and bioavailability, in specific clinical applications that are optimal for the disease context [121]. For instance, the cationic Lipoplex-based delivery of miR-29 mimics and miR-7-encoding plasmid efficiently accumulated in the tumor site and achieved significant tumor reduction in a murine xenograft tumor model of lung cancer (Figure 1a) [72,73]. Neutral liposomes have improved tumor accumulation as compared to cationic liposomes due to reduced RES clearance and prolonged circulation [122]. In fact, the neutral liposome-mediated delivery of miR-34a and let-7b mimics facilitated lung tumor-repression without elevating liver and kidney enzymes in serum without inducing a nonspecific immune response in the mice [74,75]. Ionizable liposomes (NOV340) are cationic at low pH, and neutral or anionic at neutral or higher pH, and can selectively enhance cellular uptake. This attribute was used to deliver miR-200c resulting in enhanced radiosensitivity of lung cancer in vivo [76]. The ionizable liposome-miR-34 complex (MRX34) entered phase I clinical trials for liver cancer and metastasis from other cancers (NCT01829971), which were later halted following multiple immune-related severe adverse events (SAE) observed in patients dosed with MRX34 over the course of the trial [123].

#### 3.5.2. Polymer-Based Vectors

Polymer-based vectors encompass synthetic biodegradable polymers, copolymer, or natural polymers such as collagen, chitosan and gelatin. Polymer nanoparticle’s advantages include high stability, ease of substitution, the addition of functional groups and flexibility [124]. The characteristics of these polymers such as size, morphology, charge and degradation can be altered by changing the molecular weight, polymer composition, or architecture, including linear, branched, dendrimer and copolymer. PEI, cationic polymer is the most frequently used polymer-based vector for therapeutic gene delivery. Cationic polymers have many amino groups and can use the proton sponge effect to promote escape from endosomal membranes. PEI-mediated miRNA mimics were proven effective in hepatocellular carcinoma by miR-34a, in glioblastoma by miR-145, and in colon cancer by miR-145 and -33 (Figure 1b) [77,78,79]. Cationic polymer-mediated co-delivery of miRNA mimics and chemotherapeutic drugs, such as Gemcitabine, Doxorubicin and Temozolomide, have improved therapeutic effects in in vivo tumor models [80,81,82]. While the results were promising, PEI toxicity, regardless of modifications, derived from excess positive charge, and low biological degradation in serum from non-specific binding to proteins in vivo, excluded them from a choice of clinical vectors [41].

Polyamidoamine (PAMAM) dendrimers, dendrimer-based polymeric vectors, are hyperbranched polymers with high positive surface charge, which increases the toxicity following its administration due to hepatic accumulation [125]. Conjugation with negatively charged polyethylene glycol (PEG) can prevent PAMAM toxicity [41]. Chitosan has a profound nucleic acid binding affinity when complexed with miRNA. MiR-200c in vitro and miR-34a in vivo efficiently delivered into breast cancer cells and into the bone metastasis model, respectively, resulted in decreased angiogenesis, invasion, EMT, metastasis, and increased apoptosis [126,127,128,129]. PLGA (Poly lactic-co-glycolic acid) is a negatively charged biodegradable and biocompatible polymeric nanocarrier, approved by the FDA as a drug-delivery system and is in phase II clinical trials for siRNA therapeutic delivery (NCT01676259) [110]. Physical and chemical surface manipulations, such as an addition of branched polyesters with modified amine and cationic residues (PVA-PLGA, DEAPA-PLGA, DMAPA) stabilize the nucleic acids in the polymeric matrix in order to further reduce the toxicity and degradation issues [130,131]. Indeed, the formulation of PLGA complexed with positively-charged PEI and anionic hyaluronic acid was found to efficiently deliver a DNA plasmid-containing miR-145 to colon cancer cells in vivo, thus leading to increased anti-proliferative and pro-apoptotic activities [132]. Similarly, miR-34a mimics packaged in the PLGA chitosan delivery vector decreased tumor size and improved survival in a multiple myeloma xenograft model [133]. Zhou et al. demonstrated that ester-based dendrimer carrier-mediated delivery of let-7g miRNA mimics significantly suppressed liver tumor growth with low dendrimer-related toxicity in vivo [134]. Shatsberg et al. developed a polymeric nanogel complex with miR-34a mimics for active cytoplasmic delivery, resulting in the inhibition of tumor growth in human U-87 MG GBM-bearing SCID mice, thus emphasizing the therapeutic potential of such strategy for GBM [135].

#### 3.5.3. Inorganic Vectors

Altering the size, shape, and porosity allows for the engineering of inorganic vectors. Among well-studied inorganic vectors, including calcium phosphate, porous silica, gold and carbon nanotubes, calcium phosphate (CaP) is the most successful vehicle for miRNA gene therapy. CaP, the inorganic component of bone and teeth, is biocompatible, biodegradable, easy to handle and dissolves under acidic pH in endosomes, leading to osmotic swelling and the release of encapsulated miRNA within the cell [136]. The CaP-mediated delivery of miR-4711-5p in colon cancer (Figure 1c) [84], miR-4689 in metastatic colorectal cancer [85], and miR-29b in KRAS-mutant colorectal cancer [86] were all shown to be effective. A study using a hydrogel-embedded gold-nanoparticle-based delivery system in combination with cisplatin revealed that the efficient release of miR-96 and miR-182 strongly suppressed metastasis with significant shrinkage of the primary tumor in a breast cancer mouse model [137]. In 2017, T.J. et al. showed a targeted therapy strategy for glioblastoma using folate (FA)-conjugated to three-way-junction (3WJ)-based RNA nanoparticles (RNP) for the delivery of anti-miR-21 LNA. Following its specific delivery, miR-21 expression was repressed in glioblastoma cells both in vitro and in vivo, thus resulting in antioncogenic PTEN and PDCD4 expression restoration, cell apoptosis and the regression of tumor growth [138]. Bertucci et al. developed a strategy using mesoporous silica nanoparticles (MSNPs) loaded with temozolomide (TMZ), an antineoplastic agent for malignant glial tumor treatment. These nanoparticles conjugated with the polyarginine-peptide nucleic acid R8-PNA, which targets miR-221, induced a pro-apoptotic effect in a temozolomide-resistant T98G cell line [139].

#### 3.5.4. Other Biomaterials

Atelocollagen (ATE), a nanoformulation, prepared by pepsin treatment from type I collagen of calf dermis, is one of the biomaterials with potential application as a gene delivery vector [140]. The intratumoral administration of ATE-miR-34a complex into a xenograft model of colon cancer resulted in a significant decrease in tumor size [92]. Similarly, the use of ATE in vivo for the transfection of synthetic miR-16 into the bone metastasis of prostate cancer efficiently inhibited tumor growth (Figure 1d) [93]. Additionally, the conjugation of miR-15a and miR-16-1 to ATE-RNA aptamer (APT) hybrid to target prostate-specific membrane antigen (PSMA) expressed PCa cells enhanced the strategy efficacy in vitro and in vivo by inducing the killing of cancer cells [94].

#### 3.5.5. Exosome/Extracellular Vesicle-Based Vectors

Lipid-bilayer membrane-enclosed biological nanoparticles, termed exosomes, or extracellular vesicles (EVs), are naturally secreted from cells, and mediate cellular communication within the body by transferring nucleic acids, such as DNA, RNA and proteins from one cell to the other [141,142]. Because of their potential diagnostic and therapeutic value, the exploitation of EVs for clinical application is under intensive investigation [143,144,145,146].

**Figure 2:**
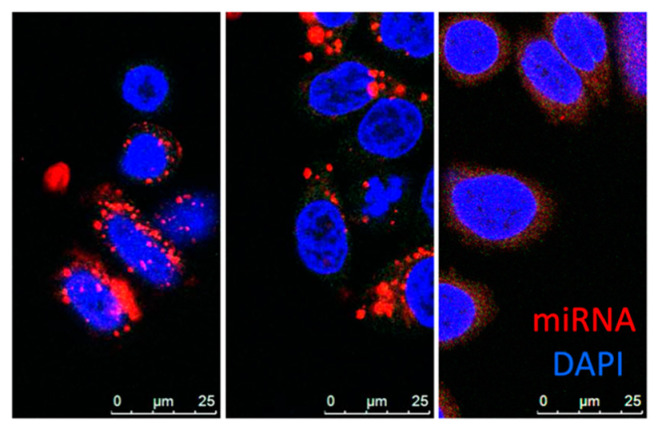
MiRNA uptake via nanoparticles. The confocal images of Dy547 labeled miRNA mimic (red) delivery to SKBR3 cells by PEI (left), lipofectamine2000 (middle), or EV (right) with DAPI staining (blue) at 24-hr time point. (Images provided by the Harada Lab).

EVs are mainly divided into three categories: exosomes, microvesicles and apoptotic bodies. Exosomes (30–150 nm) originate from the endosomal system, whereas microvesicles (50–1000 nm) shed from the plasma membrane. Despite their distinct biogenesis and intracellular origin, there is no apparent difference between these subpopulations except median size, and there is no precise method to separate these populations at this stage, thus “EV” is used as a collective term for exosomes and microvesicles of sizes around 100 nm [141,147,148,149]. As a natural biomolecular carrier, EV-mediated miRNA delivery has significant advantages over other delivery systems. MiRNAs are transported and selectively delivered to other cell types via surface receptors or proteins present on the surface of recipient cells [150,151,152,153]. Unlike synthetic nanoparticles, miRNAs delivered by EV are not entrapped by endosomes within the cell (Figure 2). Hence, the miRNAs evade the endosome trap and escape pathway, thus act faster and more efficiently in target gene repression at the cellular level. EVs can protect encapsulated miRNA from RNase degradation or harsh environments, enabling the transportation in bodily fluids [154,155]. EV-mediated miRNA delivery was proven effective for delivery of miR-143 in colon cancer [87], miR-146b in glioma [88], and miR-145 in lung cancer [89]. Other groups have demonstrated that exosome-mediated miR-122 enhanced the chemosensitivity of Sorafenib in hepatocellular carcinoma [90]. There are several methods for conjugation of peptides to EVs membrane that enhances cell specificity [155,156,157]. Ohno et al. developed EVs conjugated with epidermal growth factor (EGF)–specific peptide (GE11) that delivered let-7a to EGFR-expression breast cancer (Figure 1d) [91]. These studies revealed the promising potential of EVs to bring innovation to current therapeutic delivery approaches. However, methods of mass production, as well as effective packaging, are yet to be developed.

The advantages and disadvantages of each delivery method are shown (Table 2).

## 4. MiRNA-Based Therapies in Animal Models and Clinics

### 4.1. Therapeutic MiRNA Candidates in Preclinical Studies

Several of the miRNA-based therapeutics are currently being tested in preclinical and clinical trials. MiR-10b is a promising target for glioblastoma therapy involved in regulating cell migration, invasion and metastasis [158]. The clinical significance of miR-10b is its role in metastatic tumors where antagomiR-10b reduces metastasis in tumor-bearing mice by restoring Hoxd10 gene expression [158,159,160]. Over 100 studies on miR-10b with metastatic cancers have revealed its central role in various metastatic tumors [161]. MiR-221 is another potent target for metastatic cancer, in which anti-miR-221 caused a significant decrease in the size and number of tumor nodules in the liver of the transgenic mice model [162]. Cantafio et al. performed pharmacokinetic (PK) and pharmacodynamic (PD) studies, and revealed a short-half-life, optimal tissue bioavailability and minimal urine excretion of LNA-anti-miR-221 (LNA-i-miR-221) along with three-week span p27 target upregulation in xenograft tumors. No LNA-i-miR-221 associated toxicity was observed in their non-human primate study [163]. The loss of miR-16 is associated with a diverse range of tumors, including NSCLC [164], prostate cancer [57], or malignant pleural mesothelioma [165]. Reid et al. used bacteria-derived minicells to deliver miR-16 mimics in malignant pleural mesothelioma nude mouse models to show tumor growth inhibition through Bcl-2 and CCND1 targeting [165].

### 4.2. Clinical Studies Involving MiRNA-Based Therapy

The first siRNA drug approval for clinical use established the groundwork for miRNA drug development [166]. Mirna Therapeutics, Inc. (Carlsbad, CA, USA) developed anti-miRNA technology, including MRX34, a miR-34 mimic encapsulated in a liposomal nanoparticle formulation (NOV40). It is the first miRNA mimic to enter clinical development with a focus on patients diagnosed with primary liver cancer, NSCLC, lymphoma, melanoma, multiple myeloma, or renal cell carcinoma. In parallel, MRX34 administration alone or in combination with radiotherapy (XRT) reduced p53 regulated PDL1 expression in non-small-cell lung tumors and antagonized T-cell exhaustion. However, immune-related adverse responses led to patient deaths (immune reactions still remains unclear), after which the multicenter phase I trial was halted, and now these preclinical trials are under investigation to understand better the immune-related toxicities [55,123,167].

In collaboration with Asbestos Diseases Research Institute (Sydney, Australia), the Australian company EnGeneIC started the phase I clinical trial using miR-16 mimic (MesomiR-1) loaded to bacterial-derived nanocells system, which achieved the potent inhibition of tumor growth (NCT02369198). Malignant pleural mesothelioma (MPM) and advanced NSCLC patients, refractory to standard therapy, were intravenously administered the miR-16 mimic-based EnGeneIC Delivery Vehicle (EDV) complex in which the surface is conjugated with an EGFR-targeting antibody to facilitate tumor site targeting. The first-dose level of 5 billion nanocells loaded with 1.5 μg miR-16 mimics did not induce an adverse immune response or toxic effects, which allows for the continuation of the phase II clinical trial [168,169].

Owing to promising preclinical results, MiRagen Therapeutics, Inc. (Boulder, CO, USA) initiated the phase I clinical trial of the anticancer LNA anti-miR-155 (MRG-106) efficacy on mycosis fungoides (MF) patients, which is the most common subtype of cutaneous T-cell lymphoma (CTCL) (NCT02580552). In this trial, the reported adverse toxic effects pended the trial to optimize safer therapeutic doses and specific administration routes [41,145,170]. Table 3 shows a summary of miRNA-based Therapy Clinical Trials in the U.S.

## 5. Conclusions

The development of strategies aimed to efficiently deliver miRNAs will enhance the effectiveness of therapeutic interventions. MiRNAs are one of the most promising therapeutic targets with well-characterized expressions and functions within cancer cells with high specificity to cancer-related pathways. However, their delivery remains a challenge for miRNA therapeutics. The growing effort in the development of delivery vehicles, including nanoparticles, biomaterials and EVs, addresses the obstacles associated with cell-specific targeting, intracellular delivery, miRNA stability and carrier-associated toxicity. The newly emerged natural delivery system of exosomes/EVs offers enormous potential as a delivery vehicle in conjunction with the targeting ligand on their surface, which allows for a biocompatible delivery system with specificity to cancer cell types. The improvements in miRNA encapsulation, surface engineering and isolation/generation of EVs will advance the development of targeted-therapeutic miRNA delivery.

## Figures and Tables

**Table 1:** The Summary of MicroRNA (miRNA) Mimics or Inhibitors Tested in Vivo.

Delivery Systems	miRNAs	miRNA Type	Drug	Delivery Route	Target Disease	Target Gene	Ref
*Lipid-based system*								
Cationic liposome	miR-29b	mature-miR	-	Tail-vein	Lung cancer	CDK6, DNMT3B, MCL1	[72]
Cationic liposome	miR-7	pre-miR	-	Intratumoral	Lung cancer	IRS-1, RAF-1, EGFR	[73]
Neutral liposome	miR-34a	pre-miR	-	Intravenous	Lung cancer	BCL-2, c-Met	[74]
Neutral liposome	let-7 miR-34a	mimic-miR	-	Intravenous	Lung cancer	KRAS	[75]
Ionizable liposome	miR-200c	plasmid	-	Subcutaneous	Lung cancer	PRDX2, GAPB/Nrf2, SESN1	[76]
*Polymer-based system*								
PEI-PEG	miR-34a	dsRNA	-	Tail-vein	Hepatocellular carcinoma	SNAI1	[77]
Polyurethane-PEI	miR-145	plasmid	-	Intracranial	Glioblastoma	Oct4, Sox2	[78]
PEI	miR-145 miR-33a	dsRNA	-	Intraperitoneal Intravenous	Colon carcinoma	c-Myc, ERK5	[79]
Polymeric micelle	miR-205	mimic-miR	Gemcitabine	Intratumoral	Pancreatic cancer	ZEB-1, SIP-1, HRAS, LRP-1, CAV-1, E-CAD	[80]
Polymer micelle	anti-miR-21	dsRNA	Doxorubicin	Intratumoral	Glioma	PTEN	[81]
PACE polymer	anti-miR-21	dsRNA	Temozolomide	Intracranial	Glioblastoma	PTEN	[82]
PEI	miR-203	dsRNA	-	Subcutaneous	Basal cell carcinoma	c-JUN	[83]
*Inorganic-based system*								
Carbonate apatite	miR-4711-5p	mimic-miR	-	Intravenous	Colon cancer	KLF5, TFDP1	[84]
Carbonate apatite	miR-4689	mature-miR	-	Intravenous	Metastatic colorectal cancer	KRAS, AKT1	[85]
Carbonate apatite	miR-29b	mimic-miR	-	Intravenous	KRAS-mutant colorectal cancer	BCL-2, MCL1	[86]
*Extracellular Vesicles-based system*								
Exosome	miR-143	BP-miR		Intravenous	Colon cancer	-	[87]
Exosome	miR-146b	plasmid	-	Intratumoral	Glioma	-	[88]
Exosome	miR-145	dsRNA	-	Tail-vein	Lung cancer	CDH2	[89]
Exosome	miR-122	plasmid	Sorafenib	Intratumoral Intraperitoneal	Hepatocellular carcinoma	ADAM10, IGF1R, CCNG1	[90]
Exosome-GE11 peptide	let-7	mimic-miR	-	Intravenous	Breast Cancer	HMGA2	[91]
*Other Biomaterials*								
Atelocollagen	miR-34a	pre-miR		Subcutaneous	Colon cancer	E2F	[92]
Atelocollagen	miR-16	pre-miR		Intravenous	Prostate cancer	CDK1, CDK2	[93]
Atelocollagen	miR-15, miR-16-1	miR with 2′-fluoro		-	Prostate cancer	BCL-2, CCND1, WNT3A	[94]

**Table 2:** Advantages and Disadvantages of MicroRNA therapeutic Delivery Systems.

Delivery Systems	Advantages	Disadvantages
Viral Vectors	- High gene delivery efficiency	- Highly immunogenic
Adenoviral vector	- High gene delivery efficiency - High packaging gene-size capacity - Ability to transfer dividing cell	- Highly immunogenic - Short term transgene expression
Adeno-associated viral vector	- High gene delivery efficiency - Long term transgene expression - Organ specificity possible (serotype) - Ability to transfer dividing and non-dividing cells - Low immunogenicity	- Hard production vectors - Low gene-packaging capacity
Lentiviral vector	- High gene delivery efficiency - Long term transgene expression - Ability to transfer dividing and non-dividing cells - Low immunogenicity	- Potential genomic integration
Lipid-based system	- Ability to functionalize for targeting - Ability to co-deliver gene therapy and chemotherapy - Controllable size - Systemic gene delivery - High packaging gene-size capacity - Non-immunogenic - Transient expression	- Low delivery efficiency in vivo - Nonspecific gene delivery - Cytotoxicity
Polymer-based system	- Ability to functionalize for targeting - Ability to co-delivery gene therapy and chemotherapy - Controllable size - Systemic gene delivery - High packaging gene-size capacity - Non-immunogenic - Transient expression	- Low delivery efficiency in vivo - Nonspecific gene delivery - Cytotoxicity
Inorganic-based system	- Ability to functionalize for targeting - Controllable size - Systemic gene delivery - High packaging gene-size capacity - Non-immunogenic - Transient expression - Easy to produce	- Low gene delivery efficiency
Extracellular Vesicle-based system	- Ability to functionalize for targeting - Ability to co-delivery gene therapy and chemotherapy - Systemic gene delivery - High packaging gene-size capacity - Non-immunogenic - Highly compatibility - Low immune clearance - Organ specificity possible - High stability	- Insufficient studies on EV-based gene therapy - Diverse composition - Low production

**Table 3:** MiRNA-based Therapy Clinical Trials in the U.S.

Company	Name	Therapeutic Agent	Delivery System	Condition or Disease	Clinical Phase, Status
miRagen Therapeutics	MRG-106	Anti-miR-155	LNA-antisense	Cutaneous T-cell Lymphoma (CTCL)/Mycosis Fungoides (MF)	Phase II (NCT03837457), Enrolling by invitation
					Phase I (NCT03713320), Active, not recruiting
miRagen Therapeutics	MRG-106	Anti-miR-155	LNA-antisense	CTCL, MF, Chronic Lymphocytic Leukemia (CLL), Diffuse Large B-cell Lymphoma (DVBCL), Activated B-cell (ABC) subtype, Adult T-cell Leukemia/Lymphoma (ATLL)	Phase I (NCT02580552), Active, not recruiting
Mirna Therapeutics Inc.	MRX-34	miR-34 mimic	dsRNA liposomal nanoparticle	Melanoma	Phase I (NCT02862145), Withdrawn
Mirna Therapeutics Inc.	MRX-34	miR-34 mimic	dsRNA liposomal nanoparticle	Primary liver cancer, SCLS, Lymphoma, Melanoma, Multiple myeloma, Renal cell carcinoma, NSCLC	Phase I (NCT01829971), Terminated
EnGeneIC	MesomiR-1	miR-16 mimic	EnGeneIC Dream Vector	Malignant pleural mesothelioma, Non-small-cell lung cancer	Phase I (NCT02369198), Completed

## References

[1] Liu Y., Corcoran M., Rasool O., Ivanova G., Ibbotson R., Grander D., Iyengar A., Baranova A., Kashuba V., Merup M. (1997). Cloning of two candidate tumor suppressor genes within a 10 kb region on chromosome 13q14, frequently deleted in chronic lymphocytic leukemia. Oncogene.

[2] Calin G.A., Dumitru C.D., Shimizu M., Bichi R., Zupo S., Noch E., Aldler H., Rattan S., Keating M., Rai K. (2002). Frequent deletions and down-regulation of micro- RNA genes miR15 and miR16 at 13q14 in chronic lymphocytic leukemia. Proc. Natl. Acad. Sci. USA.

[3] Cimmino A., Calin G.A., Fabbri M., Iorio M.V., Ferracin M., Shimizu M., Wojcik S.E., Aqeilan R.I., Zupo S., Dono M. (2005). miR-15 and miR-16 induce apoptosis by targeting BCL2. Proc. Natl. Acad. Sci. USA.

[4] Lerner M., Harada M., Loven J., Castro J., Davis Z., Oscier D., Henriksson M., Sangfelt O., Grander D., Corcoran M.M. (2009). DLEU2, frequently deleted in malignancy, functions as a critical host gene of the cell cycle inhibitory microRNAs miR-15a and miR-16-1. Exp. Cell Res..

[5] Cai X., Hagedorn C.H., Cullen B.R. (2004). Human microRNAs are processed from capped, polyadenylated transcripts that can also function as mRNAs. RNA.

[6] Lee Y., Ahn C., Han J., Choi H., Kim J., Yim J., Lee J., Provost P., Radmark O., Kim S. (2003). The nuclear RNase III Drosha initiates microRNA processing. Nature.

[7] Hutvagner G., McLachlan J., Pasquinelli A.E., Balint E., Tuschl T., Zamore P.D. (2001). A cellular function for the RNA-interference enzyme Dicer in the maturation of the let-7 small temporal RNA. Science.

[8] Cifuentes D., Xue H., Taylor D.W., Patnode H., Mishima Y., Cheloufi S., Ma E., Mane S., Hannon G.J., Lawson N.D. (2010). A novel miRNA processing pathway independent of Dicer requires Argonaute2 catalytic activity. Science.

[9] Ha M., Kim V.N. (2014). Regulation of microRNA biogenesis. Nat. Rev. Mol. Cell Biol..

[10] Treiber T., Treiber N., Meister G. (2019). Regulation of microRNA biogenesis and its crosstalk with other cellular pathways. Nat. Rev. Mol. Cell Biol..

[11] Merritt W.M., Lin Y.G., Han L.Y., Kamat A.A., Spannuth W.A., Schmandt R., Urbauer D., Pennacchio L.A., Cheng J.F., Nick A.M. (2008). Dicer, Drosha, and outcomes in patients with ovarian cancer. N. Engl. J. Med..

[12] Lin R.J., Lin Y.C., Chen J., Kuo H.H., Chen Y.Y., Diccianni M.B., London W.B., Chang C.H., Yu A.L. (2010). microRNA signature and expression of Dicer and Drosha can predict prognosis and delineate risk groups in neuroblastoma. Cancer Res..

[13] Karube Y., Tanaka H., Osada H., Tomida S., Tatematsu Y., Yanagisawa K., Yatabe Y., Takamizawa J., Miyoshi S., Mitsudomi T. (2005). Reduced expression of Dicer associated with poor prognosis in lung cancer patients. Cancer Sci..

[14] Lewis B.P., Burge C.B., Bartel D.P. (2005). Conserved seed pairing, often flanked by adenosines, indicates that thousands of human genes are microRNA targets. Cell.

[15] Friedman R.C., Farh K.K., Burge C.B., Bartel D.P. (2009). Most mammalian mRNAs are conserved targets of microRNAs. Genome Res..

[16] Ameres S.L., Zamore P.D. (2013). Diversifying microRNA sequence and function. Nat. Rev. Mol. Cell Biol..

[17] Hinske L.C., Franca G.S., Torres H.A., Ohara D.T., Lopes-Ramos C.M., Heyn J., Reis L.F., Ohno-Machado L., Kreth S., Galante P.A. (2014). miRIAD-integrating microRNA inter- and intragenic data. Database (Oxf.).

[18] Misiewicz-Krzeminska I., Krzeminski P., Corchete L.A., Quwaider D., Rojas E.A., Herrero A.B., Gutierrez N.C. (2019). Factors Regulating microRNA Expression and Function in Multiple Myeloma. Noncoding RNA.

[19] Gulyaeva L.F., Kushlinskiy N.E. (2016). Regulatory mechanisms of microRNA expression. J. Transl. Med..

[20] Bail S., Swerdel M., Liu H., Jiao X., Goff L.A., Hart R.P., Kiledjian M. (2010). Differential regulation of microRNA stability. RNA.

[21] Harada M., Pokrovskaja-Tamm K., Soderhall S., Heyman M., Grander D., Corcoran M. (2012). Involvement of miR17 pathway in glucocorticoid-induced cell death in pediatric acute lymphoblastic leukemia. Leuk Lymphoma.

[22] Lin S., Gregory R.I. (2015). MicroRNA biogenesis pathways in cancer. Nat. Rev. Cancer.

[23] Whiteside T.L. (2008). The tumor microenvironment and its role in promoting tumor growth. Oncogene.

[24] Bhome R., Goh R.W., Bullock M.D., Pillar N., Thirdborough S.M., Mellone M., Mirnezami R., Galea D., Veselkov K., Gu Q. (2017). Exosomal microRNAs derived from colorectal cancer-associated fibroblasts: Role in driving cancer progression. Aging (Albany NY).

[25] Donnarumma E., Fiore D., Nappa M., Roscigno G., Adamo A., Iaboni M., Russo V., Affinito A., Puoti I., Quintavalle C. (2017). Cancer-associated fibroblasts release exosomal microRNAs that dictate an aggressive phenotype in breast cancer. Oncotarget.

[26] Richards K.E., Zeleniak A.E., Fishel M.L., Wu J., Littlepage L.E., Hill R. (2017). Cancer-associated fibroblast exosomes regulate survival and proliferation of pancreatic cancer cells. Oncogene.

[27] Au Yeung C.L., Co N.N., Tsuruga T., Yeung T.L., Kwan S.Y., Leung C.S., Li Y., Lu E.S., Kwan K., Wong K.K. (2016). Exosomal transfer of stroma-derived miR21 confers paclitaxel resistance in ovarian cancer cells through targeting APAF1. Nat. Commun..

[28] Kohlhapp F.J., Mitra A.K., Lengyel E., Peter M.E. (2015). MicroRNAs as mediators and communicators between cancer cells and the tumor microenvironment. Oncogene.

[29] Berindan-Neagoe I., Monroig P.e.C., Pasculli B., Calin G.A. (2014). MicroRNAome genome: A treasure for cancer diagnosis and therapy. CA Cancer J. Clin..

[30] Di Leva G., Garofalo M., Croce C.M. (2014). MicroRNAs in cancer. Annu. Rev. Pathol..

[31] Shah M.Y., Ferrajoli A., Sood A.K., Lopez-Berestein G., Calin G.A. (2016). microRNA Therapeutics in Cancer-An Emerging Concept. EBioMedicine.

[32] Lan H., Lu H., Wang X., Jin H. (2015). MicroRNAs as potential biomarkers in cancer: Opportunities and challenges. Biomed. Res. Int..

[33] Lu J., Getz G., Miska E.A., Alvarez-Saavedra E., Lamb J., Peck D., Sweet-Cordero A., Ebert B.L., Mak R.H., Ferrando A.A. (2005). MicroRNA expression profiles classify human cancers. Nature.

[34] Zhang B., Pan X., Cobb G.P., Anderson T.A. (2007). microRNAs as oncogenes and tumor suppressors. Dev. Biol..

[35] Iqbal J., Shen Y., Huang X., Liu Y., Wake L., Liu C., Deffenbacher K., Lachel C.M., Wang C., Rohr J. (2015). Global microRNA expression profiling uncovers molecular markers for classification and prognosis in aggressive B-cell lymphoma. Blood.

[36] Caramuta S., Lee L., Ozata D.M., Akçakaya P., Georgii-Hemming P., Xie H., Amini R.M., Lawrie C.H., Enblad G., Larsson C. (2013). Role of microRNAs and microRNA machinery in the pathogenesis of diffuse large B-cell lymphoma. Blood Cancer J..

[37] Xiao C., Calado D.P., Galler G., Thai T.H., Patterson H.C., Wang J., Rajewsky N., Bender T.P., Rajewsky K. (2007). MiR-150 controls B cell differentiation by targeting the transcription factor c-Myb. Cell.

[38] Li Y., Cai B., Shen L., Dong Y., Lu Q., Sun S., Liu S., Ma S., Ma P.X., Chen J. (2017). MiRNA-29b suppresses tumor growth through simultaneously inhibiting angiogenesis and tumorigenesis by targeting Akt3. Cancer Lett..

[39] Croce C.M. (2009). Causes and consequences of microRNA dysregulation in cancer. Nat. Rev. Genet..

[40] Esquela-Kerscher A., Slack F.J. (2006). Oncomirs-microRNAs with a role in cancer. Nat. Rev. Cancer.

[41] Hosseinahli N., Aghapour M., Duijf P.H.G., Baradaran B. (2018). Treating cancer with microRNA replacement therapy: A literature review. J. Cell Physiol..

[42] Osaki M., Takeshita F., Sugimoto Y., Kosaka N., Yamamoto Y., Yoshioka Y., Kobayashi E., Yamada T., Kawai A., Inoue T. (2011). MicroRNA-143 regulates human osteosarcoma metastasis by regulating matrix metalloprotease-13 expression. Mol. Ther..

[43] Qiu T., Zhou X., Wang J., Du Y., Xu J., Huang Z., Zhu W., Shu Y., Liu P. (2014). MiR-145, miR-133a and miR-133b inhibit proliferation, migration, invasion and cell cycle progression via targeting transcription factor Sp1 in gastric cancer. FEBS Lett..

[44] Bader A.G., Brown D., Winkler M. (2010). The promise of microRNA replacement therapy. Cancer Res..

[45] Broderick J.A., Zamore P.D. (2011). MicroRNA therapeutics. Gene Ther..

[46] Kong Y.W., Ferland-McCollough D., Jackson T.J., Bushell M. (2012). microRNAs in cancer management. Lancet Oncol..

[47] Lu Z., He Q., Liang J., Li W., Su Q., Chen Z., Wan Q., Zhou X., Cao L., Sun J. (2019). miR-31-5p Is a Potential Circulating Biomarker and Therapeutic Target for Oral Cancer. Mol. Ther. Nucleic Acids.

[48] Kao Y.Y., Chou C.H., Yeh L.Y., Chen Y.F., Chang K.W., Liu C.J., Fan Chiang C.Y., Lin S.C. (2019). MicroRNA miR-31 targets SIRT3 to disrupt mitochondrial activity and increase oxidative stress in oral carcinoma. Cancer Lett..

[49] Guo H., Ji F., Zhao X., Yang X., He J., Huang L., Zhang Y. (2019). MicroRNA-371a-3p promotes progression of gastric cancer by targeting TOB1. Cancer Lett..

[50] Dieckmann K.P., Spiekermann M., Balks T., Flor I., Löning T., Bullerdiek J., Belge G. (2012). MicroRNAs miR-371-3 in serum as diagnostic tools in the management of testicular germ cell tumours. Br. J. Cancer.

[51] Yanaihara N., Caplen N., Bowman E., Seike M., Kumamoto K., Yi M., Stephens R.M., Okamoto A., Yokota J., Tanaka T. (2006). Unique microRNA molecular profiles in lung cancer diagnosis and prognosis. Cancer Cell.

[52] Kong Y., Zou S., Yang F., Xu X., Bu W., Jia J., Liu Z. (2016). RUNX3-mediated up-regulation of miR-29b suppresses the proliferation and migration of gastric cancer cells by targeting KDM2A. Cancer Lett..

[53] Yang S., Li Y., Gao J., Zhang T., Li S., Luo A., Chen H., Ding F., Wang X., Liu Z. (2013). MicroRNA-34 suppresses breast cancer invasion and metastasis by directly targeting Fra-1. Oncogene.

[54] Ma W., Xiao G.G., Mao J., Lu Y., Song B., Wang L., Fan S., Fan P., Hou Z., Li J. (2015). Dysregulation of the miR-34a-SIRT1 axis inhibits breast cancer stemness. Oncotarget.

[55] Beg M.S., Brenner A.J., Sachdev J., Borad M., Kang Y.K., Stoudemire J., Smith S., Bader A.G., Kim S., Hong D.S. (2017). Phase I study of MRX34, a liposomal miR-34a mimic, administered twice weekly in patients with advanced solid tumors. Invest. New Drugs.

[56] Zhang X.J., Ye H., Zeng C.W., He B., Zhang H., Chen Y.Q. (2010). Dysregulation of miR-15a and miR-214 in human pancreatic cancer. J. Hematol. Oncol..

[57] Bonci D., Coppola V., Musumeci M., Addario A., Giuffrida R., Memeo L., D’Urso L., Pagliuca A., Biffoni M., Labbaye C. (2008). The miR-15a-miR-16-1 cluster controls prostate cancer by targeting multiple oncogenic activities. Nat. Med..

[58] Elmén J., Lindow M., Schütz S., Lawrence M., Petri A., Obad S., Lindholm M., Hedtjärn M., Hansen H.F., Berger U. (2008). LNA-mediated microRNA silencing in non-human primates. Nature.

[59] Garzon R., Marcucci G., Croce C.M. (2010). Targeting microRNAs in cancer: Rationale, strategies and challenges. Nat. Rev. Drug Discov..

[60] Monroig P.e.C., Chen L., Zhang S., Calin G.A. (2015). Small molecule compounds targeting miRNAs for cancer therapy. Adv. Drug Deliv. Rev..

[61] Vester B., Wengel J. (2004). LNA (locked nucleic acid): High-affinity targeting of complementary RNA and DNA. Biochemistry.

[62] Krützfeldt J., Rajewsky N., Braich R., Rajeev K.G., Tuschl T., Manoharan M., Stoffel M. (2005). Silencing of microRNAs in vivo with ‘antagomirs’. Nature.

[63] Han M., Liu M., Wang Y., Chen X., Xu J., Sun Y., Zhao L., Qu H., Fan Y., Wu C. (2012). Antagonism of miR-21 reverses epithelial-mesenchymal transition and cancer stem cell phenotype through AKT/ERK1/2 inactivation by targeting PTEN. PLoS ONE.

[64] Liu L.Z., Li C., Chen Q., Jing Y., Carpenter R., Jiang Y., Kung H.F., Lai L., Jiang B.H. (2011). MiR-21 induced angiogenesis through AKT and ERK activation and HIF-1α expression. PLoS ONE.

[65] Chen Y., Gao D.Y., Huang L. (2015). In vivo delivery of miRNAs for cancer therapy: Challenges and strategies. Adv. Drug Deliv. Rev..

[66] Chen L., Zhang K., Shi Z., Zhang A., Jia Z., Wang G., Pu P., Kang C., Han L. (2014). A lentivirus-mediated miR-23b sponge diminishes the malignant phenotype of glioma cells in vitro and in vivo. Oncol. Rep..

[67] Jansson M.D., Lund A.H. (2012). MicroRNA and cancer. Mol. Oncol..

[68] Kota J., Chivukula R.R., O’Donnell K.A., Wentzel E.A., Montgomery C.L., Hwang H.W., Chang T.C., Vivekanandan P., Torbenson M., Clark K.R. (2009). Therapeutic microRNA delivery suppresses tumorigenesis in a murine liver cancer model. Cell.

[69] Stylianopoulos T., Jain R.K. (2013). Combining two strategies to improve perfusion and drug delivery in solid tumors. Proc. Natl. Acad. Sci. USA.

[70] Kleger A., Perkhofer L., Seufferlein T. (2014). Smarter drugs emerging in pancreatic cancer therapy. Ann. Oncol..

[71] Castelli D.D., Terreno E., Cabella C., Chaabane L., Lanzardo S., Tei L., Visigalli M., Aime S. (2009). Evidence for in vivo macrophage mediated tumor uptake of paramagnetic/fluorescent liposomes. NMR Biomed..

[72] Wu Y., Crawford M., Mao Y., Lee R.J., Davis I.C., Elton T.S., Lee L.J., Nana-Sinkam S.P. (2013). Therapeutic Delivery of MicroRNA-29b by Cationic Lipoplexes for Lung Cancer. Mol. Ther. Nucleic Acids.

[73] Rai K., Takigawa N., Ito S., Kashihara H., Ichihara E., Yasuda T., Shimizu K., Tanimoto M., Kiura K. (2011). Liposomal delivery of MicroRNA-7-expressing plasmid overcomes epidermal growth factor receptor tyrosine kinase inhibitor-resistance in lung cancer cells. Mol. Cancer Ther..

[74] Wiggins J.F., Ruffino L., Kelnar K., Omotola M., Patrawala L., Brown D., Bader A.G. (2010). Development of a lung cancer therapeutic based on the tumor suppressor microRNA-34. Cancer Res..

[75] Trang P., Wiggins J.F., Daige C.L., Cho C., Omotola M., Brown D., Weidhaas J.B., Bader A.G., Slack F.J. (2011). Systemic delivery of tumor suppressor microRNA mimics using a neutral lipid emulsion inhibits lung tumors in mice. Mol. Ther..

[76] Cortez M.A., Valdecanas D., Zhang X., Zhan Y., Bhardwaj V., Calin G.A., Komaki R., Giri D.K., Quini C.C., Wolfe T. (2014). Therapeutic delivery of miR-200c enhances radiosensitivity in lung cancer. Mol. Ther..

[77] Hu Q., Wang K., Sun X., Li Y., Fu Q., Liang T., Tang G. (2016). A redox-sensitive, oligopeptide-guided, self-assembling, and efficiency-enhanced (ROSE) system for functional delivery of microRNA therapeutics for treatment of hepatocellular carcinoma. Biomaterials.

[78] Yang Y.P., Chien Y., Chiou G.Y., Cherng J.Y., Wang M.L., Lo W.L., Chang Y.L., Huang P.I., Chen Y.W., Shih Y.H. (2012). Inhibition of cancer stem cell-like properties and reduced chemoradioresistance of glioblastoma using microRNA145 with cationic polyurethane-short branch PEI. Biomaterials.

[79] Ibrahim A.F., Weirauch U., Thomas M., Grünweller A., Hartmann R.K., Aigner A. (2011). MicroRNA replacement therapy for miR-145 and miR-33a is efficacious in a model of colon carcinoma. Cancer Res..

[80] Mittal A., Chitkara D., Behrman S.W., Mahato R.I. (2014). Efficacy of gemcitabine conjugated and miRNA-205 complexed micelles for treatment of advanced pancreatic cancer. Biomaterials.

[81] Qian X., Long L., Shi Z., Liu C., Qiu M., Sheng J., Pu P., Yuan X., Ren Y., Kang C. (2014). Star-branched amphiphilic PLA-b-PDMAEMA copolymers for co-delivery of miR-21 inhibitor and doxorubicin to treat glioma. Biomaterials.

[82] Seo Y.E., Suh H.W., Bahal R., Josowitz A., Zhang J., Song E., Cui J., Noorbakhsh S., Jackson C., Bu T. (2019). Nanoparticle-mediated intratumoral inhibition of miR-21 for improved survival in glioblastoma. Biomaterials.

[83] Sonkoly E., Lovén J., Xu N., Meisgen F., Wei T., Brodin P., Jaks V., Kasper M., Shimokawa T., Harada M. (2012). MicroRNA-203 functions as a tumor suppressor in basal cell carcinoma. Oncogenesis.

[84] Morimoto Y., Mizushima T., Wu X., Okuzaki D., Yokoyama Y., Inoue A., Hata T., Hirose H., Qian Y., Wang J. (2020). miR-4711-5p regulates cancer stemness and cell cycle progression via KLF5, MDM2 and TFDP1 in colon cancer cells. Br. J. Cancer.

[85] Hiraki M., Nishimura J., Takahashi H., Wu X., Takahashi Y., Miyo M., Nishida N., Uemura M., Hata T., Takemasa I. (2015). Concurrent Targeting of KRAS and AKT by MiR-4689 Is a Novel Treatment Against Mutant KRAS Colorectal Cancer. Mol. Ther. Nucleic Acids.

[86] Inoue A., Mizushima T., Wu X., Okuzaki D., Kambara N., Ishikawa S., Wang J., Qian Y., Hirose H., Yokoyama Y. (2018). A miR-29b Byproduct Sequence Exhibits Potent Tumor-Suppressive Activities via Inhibition of NF-κB Signaling in. Mol. Cancer Ther..

[87] Akao Y., Iio A., Itoh T., Noguchi S., Itoh Y., Ohtsuki Y., Naoe T. (2011). Microvesicle-mediated RNA molecule delivery system using monocytes/macrophages. Mol. Ther..

[88] Katakowski M., Buller B., Zheng X., Lu Y., Rogers T., Osobamiro O., Shu W., Jiang F., Chopp M. (2013). Exosomes from marrow stromal cells expressing miR-146b inhibit glioma growth. Cancer Lett..

[89] Vázquez-Ríos A.J., Molina-Crespo Á., Bouzo B.L., López-López R., Moreno-Bueno G., de la Fuente M. (2019). Exosome-mimetic nanoplatforms for targeted cancer drug delivery. J. Nanobiotechnol..

[90] Lou G., Song X., Yang F., Wu S., Wang J., Chen Z., Liu Y. (2015). Exosomes derived from miR-122-modified adipose tissue-derived MSCs increase chemosensitivity of hepatocellular carcinoma. J. Hematol. Oncol..

[91] Ohno S., Takanashi M., Sudo K., Ueda S., Ishikawa A., Matsuyama N., Fujita K., Mizutani T., Ohgi T., Ochiya T. (2013). Systemically injected exosomes targeted to EGFR deliver antitumor microRNA to breast cancer cells. Mol. Ther..

[92] Tazawa H., Tsuchiya N., Izumiya M., Nakagama H. (2007). Tumor-suppressive miR-34a induces senescence-like growth arrest through modulation of the E2F pathway in human colon cancer cells. Proc. Natl. Acad. Sci. USA.

[93] Takeshita F., Patrawala L., Osaki M., Takahashi R.U., Yamamoto Y., Kosaka N., Kawamata M., Kelnar K., Bader A.G., Brown D. (2010). Systemic delivery of synthetic microRNA-16 inhibits the growth of metastatic prostate tumors via downregulation of multiple cell-cycle genes. Mol. Ther..

[94] Hao Z., Fan W., Hao J., Wu X., Zeng G.Q., Zhang L.J., Nie S.F., Wang X.D. (2016). Efficient delivery of micro RNA to bone-metastatic prostate tumors by using aptamer-conjugated atelocollagen in vitro and in vivo. Drug Deliv..

[95] Halle B., Marcusson E.G., Aaberg-Jessen C., Jensen S.S., Meyer M., Schulz M.K., Andersen C., Kristensen B.W. (2016). Convection-enhanced delivery of an anti-miR is well-tolerated, preserves anti-miR stability and causes efficient target de-repression: A proof of concept. J. Neurooncol..

[96] Trang P., Medina P.P., Wiggins J.F., Ruffino L., Kelnar K., Omotola M., Homer R., Brown D., Bader A.G., Weidhaas J.B. (2010). Regression of murine lung tumors by the let-7 microRNA. Oncogene.

[97] He X.X., Chang Y., Meng F.Y., Wang M.Y., Xie Q.H., Tang F., Li P.Y., Song Y.H., Lin J.S. (2012). MicroRNA-375 targets AEG-1 in hepatocellular carcinoma and suppresses liver cancer cell growth in vitro and in vivo. Oncogene.

[98] Sureban S.M., May R., Mondalek F.G., Qu D., Ponnurangam S., Pantazis P., Anant S., Ramanujam R.P., Houchen C.W. (2011). Nanoparticle-based delivery of siDCAMKL-1 increases microRNA-144 and inhibits colorectal cancer tumor growth via a Notch-1 dependent mechanism. J. Nanobiotechnol..

[99] Aagaard L., Rossi J.J. (2007). RNAi therapeutics: Principles, prospects and challenges. Adv. Drug Deliv. Rev..

[100] Wang X., Yu B., Ren W., Mo X., Zhou C., He H., Jia H., Wang L., Jacob S.T., Lee R.J. (2013). Enhanced hepatic delivery of siRNA and microRNA using oleic acid based lipid nanoparticle formulations. J. Control. Release.

[101] Davis S., Lollo B., Freier S., Esau C. (2006). Improved targeting of miRNA with antisense oligonucleotides. Nucleic Acids Res..

[102] Elmén J., Lindow M., Silahtaroglu A., Bak M., Christensen M., Lind-Thomsen A., Hedtjärn M., Hansen J.B., Hansen H.F., Straarup E.M. (2008). Antagonism of microRNA-122 in mice by systemically administered LNA-antimiR leads to up-regulation of a large set of predicted target mRNAs in the liver. Nucleic Acids Res..

[103] Stenvang J., Petri A., Lindow M., Obad S., Kauppinen S. (2012). Inhibition of microRNA function by antimiR oligonucleotides. Silence.

[104] Teplyuk N.M., Uhlmann E.J., Gabriely G., Volfovsky N., Wang Y., Teng J., Karmali P., Marcusson E., Peter M., Mohan A. (2016). Therapeutic potential of targeting microRNA-10b in established intracranial glioblastoma: First steps toward the clinic. EMBO Mol. Med..

[105] Yang N. (2015). An overview of viral and nonviral delivery systems for microRNA. Int. J. Pharm. Investig..

[106] O’Neill C.P., Dwyer R.M. (2020). Nanoparticle-Based Delivery of Tumor Suppressor microRNA for Cancer Therapy. Cells.

[107] Herrera-Carrillo E., Liu Y.P., Berkhout B. (2017). Improving miRNA Delivery by Optimizing miRNA Expression Cassettes in Diverse Virus Vectors. Hum. Gene Ther. Methods.

[108] Kasar S., Salerno E., Yuan Y., Underbayev C., Vollenweider D., Laurindo M.F., Fernandes H., Bonci D., Addario A., Mazzella F. (2012). Systemic in vivo lentiviral delivery of miR-15a/16 reduces malignancy in the NZB de novo mouse model of chronic lymphocytic leukemia. Genes Immun..

[109] Liu Y., Lai L., Chen Q., Song Y., Xu S., Ma F., Wang X., Wang J., Yu H., Cao X. (2012). MicroRNA-494 is required for the accumulation and functions of tumor-expanded myeloid-derived suppressor cells via targeting of PTEN. J. Immunol..

[110] Yin H., Kanasty R.L., Eltoukhy A.A., Vegas A.J., Dorkin J.R., Anderson D.G. (2014). Non-viral vectors for gene-based therapy. Nat. Rev. Genet..

[111] Bai Z., Wei J., Yu C., Han X., Qin X., Zhang C., Liao W., Li L., Huang W. (2019). Non-viral nanocarriers for intracellular delivery of microRNA therapeutics. J. Mater. Chem. B.

[112] Wang H., Liu S., Jia L., Chu F., Zhou Y., He Z., Guo M., Chen C., Xu L. (2018). Nanostructured lipid carriers for MicroRNA delivery in tumor gene therapy. Cancer Cell Int..

[113] Bakhshandeh B., Soleimani M., Hafizi M., Ghaemi N. (2012). A comparative study on nonviral genetic modifications in cord blood and bone marrow mesenchymal stem cells. Cytotechnology.

[114] Vaughan H.J., Green J.J., Tzeng S.Y. (2020). Cancer-Targeting Nanoparticles for Combinatorial Nucleic Acid Delivery. Adv. Mater..

[115] Mintzer M.A., Simanek E.E. (2009). Nonviral vectors for gene delivery. Chem. Rev..

[116] Wang A.Z., Langer R., Farokhzad O.C. (2012). Nanoparticle delivery of cancer drugs. Annu. Rev. Med..

[117] Pecot C.V., Calin G.A., Coleman R.L., Lopez-Berestein G., Sood A.K. (2011). RNA interference in the clinic: Challenges and future directions. Nat. Rev. Cancer.

[118] Blanco E., Shen H., Ferrari M. (2015). Principles of nanoparticle design for overcoming biological barriers to drug delivery. Nat. Biotechnol..

[119] Cabral H., Matsumoto Y., Mizuno K., Chen Q., Murakami M., Kimura M., Terada Y., Kano M., Miyazono K., Uesaka M. (2011). Accumulation of sub-100 nm polymeric micelles in poorly permeable tumours depends on size. Nat. Nanotechnol..

[120] Torchilin V.P. (2005). Recent advances with liposomes as pharmaceutical carriers. Nat. Rev. Drug Discov..

[121] Ait-Oudhia S., Mager D.E., Straubinger R.M. (2014). Application of pharmacokinetic and pharmacodynamic analysis to the development of liposomal formulations for oncology. Pharmaceutics.

[122] Sun X., Yan X., Jacobson O., Sun W., Wang Z., Tong X., Xia Y., Ling D., Chen X. (2017). Improved Tumor Uptake by Optimizing Liposome Based RES Blockade Strategy. Theranostics.

[123] Hong D.S., Kang Y.K., Borad M., Sachdev J., Ejadi S., Lim H.Y., Brenner A.J., Park K., Lee J.L., Kim T.Y. (2020). Phase 1 study of MRX34, a liposomal miR-34a mimic, in patients with advanced solid tumours. Br. J. Cancer.

[124] Fernandez C.A., Rice K.G. (2009). Engineered nanoscaled polyplex gene delivery systems. Mol. Pharm..

[125] Duncan R., Izzo L. (2005). Dendrimer biocompatibility and toxicity. Adv. Drug Deliv. Rev..

[126] Mao S., Sun W., Kissel T. (2010). Chitosan-based formulations for delivery of DNA and siRNA. Adv. Drug Deliv. Rev..

[127] Kaban K., Salva E., Akbuga J. (2016). The effects of chitosan/miR-200c nanoplexes on different stages of cancers in breast cancer cell lines. Eur. J. Pharm. Sci..

[128] Kaban K., Salva E., Akbuga J. (2017). In Vitro Dose Studies on Chitosan Nanoplexes for microRNA Delivery in Breast Cancer Cells. Nucleic Acid Ther..

[129] Gaur S., Wen Y., Song J.H., Parikh N.U., Mangala L.S., Blessing A.M., Ivan C., Wu S.Y., Varkaris A., Shi Y. (2015). Chitosan nanoparticle-mediated delivery of miRNA-34a decreases prostate tumor growth in the bone and its expression induces non-canonical autophagy. Oncotarget.

[130] Dailey L.A., Wittmar M., Kissel T. (2005). The role of branched polyesters and their modifications in the development of modern drug delivery vehicles. J. Control. Release.

[131] Tinsley-Bown A.M., Fretwell R., Dowsett A.B., Davis S.L., Farrar G.H. (2000). Formulation of poly(D,L-lactic-co-glycolic acid) microparticles for rapid plasmid DNA delivery. J. Control. Release.

[132] Liang G., Zhu Y., Jing A., Wang J., Hu F., Feng W., Xiao Z., Chen B. (2016). Cationic microRNA-delivering nanocarriers for efficient treatment of colon carcinoma in xenograft model. Gene Ther..

[133] Cosco D., Cilurzo F., Maiuolo J., Federico C., Di Martino M.T., Cristiano M.C., Tassone P., Fresta M., Paolino D. (2015). Delivery of miR-34a by chitosan/PLGA nanoplexes for the anticancer treatment of multiple myeloma. Sci. Rep..

[134] Zhou K., Nguyen L.H., Miller J.B., Yan Y., Kos P., Xiong H., Li L., Hao J., Minnig J.T., Zhu H. (2016). Modular degradable dendrimers enable small RNAs to extend survival in an aggressive liver cancer model. Proc. Natl. Acad. Sci. USA.

[135] Shatsberg Z., Zhang X., Ofek P., Malhotra S., Krivitsky A., Scomparin A., Tiram G., Calderón M., Haag R., Satchi-Fainaro R. (2016). Functionalized nanogels carrying an anticancer microRNA for glioblastoma therapy. J. Control. Release.

[136] Sokolova V., Epple M. (2008). Inorganic nanoparticles as carriers of nucleic acids into cells. Angew. Chem. Int. Ed. Engl..

[137] Gilam A., Conde J., Weissglas-Volkov D., Oliva N., Friedman E., Artzi N., Shomron N. (2016). Local microRNA delivery targets Palladin and prevents metastatic breast cancer. Nat. Commun..

[138] Lee T.J., Yoo J.Y., Shu D., Li H., Zhang J., Yu J.G., Jaime-Ramirez A.C., Acunzo M., Romano G., Cui R. (2017). RNA Nanoparticle-Based Targeted Therapy for Glioblastoma through Inhibition of Oncogenic miR-21. Mol. Ther..

[139] Bertucci A., Prasetyanto E.A., Septiadi D., Manicardi A., Brognara E., Gambari R., Corradini R., De Cola L. (2015). Combined Delivery of Temozolomide and Anti-miR221 PNA Using Mesoporous Silica Nanoparticles Induces Apoptosis in Resistant Glioma Cells. Small.

[140] Ochiya T., Nagahara S., Sano A., Itoh H., Terada M. (2001). Biomaterials for gene delivery: Atelocollagen-mediated controlled release of molecular medicines. Curr. Gene Ther..

[141] Tkach M., Théry C. (2016). Communication by Extracellular Vesicles: Where We Are and Where We Need to Go. Cell.

[142] Simpson R.J., Jensen S.S., Lim J.W. (2008). Proteomic profiling of exosomes: Current perspectives. Proteomics.

[143] EL Andaloussi S., Mäger I., Breakefield X.O., Wood M.J. (2013). Extracellular vesicles: Biology and emerging therapeutic opportunities. Nat. Rev. Drug Discov..

[144] Fuhrmann G., Herrmann I., Stevens M. (2015). Cell-derived vesicles for drug therapy and diagnostics: Opportunities and challenges. Nano Today.

[145] Rupaimoole R., Slack F.J. (2017). MicroRNA therapeutics: Towards a new era for the management of cancer and other diseases. Nat. Rev. Drug Discov..

[146] Kalluri R., LeBleu V.S. (2020). The biology, function, and biomedical applications of exosomes. Science.

[147] Hessvik N.P., Llorente A. (2018). Current knowledge on exosome biogenesis and release. Cell Mol. Life Sci..

[148] Stahl P.D., Raposo G. (2018). Exosomes and extracellular vesicles: The path forward. Essays Biochem..

[149] Thery C., Witwer K.W., Aikawa E., Alcaraz M.J., Anderson J.D., Andriantsitohaina R., Antoniou A., Arab T., Archer F., Atkin-Smith G.K. (2018). Minimal information for studies of extracellular vesicles 2018 (MISEV2018): A position statement of the International Society for Extracellular Vesicles and update of the MISEV2014 guidelines. J. Extracell Vesicles.

[150] Valadi H., Ekström K., Bossios A., Sjöstrand M., Lee J.J., Lötvall J.O. (2007). Exosome-mediated transfer of mRNAs and microRNAs is a novel mechanism of genetic exchange between cells. Nat. Cell Biol..

[151] Skog J., Würdinger T., van Rijn S., Meijer D.H., Gainche L., Sena-Esteves M., Curry W.T., Carter B.S., Krichevsky A.M., Breakefield X.O. (2008). Glioblastoma microvesicles transport RNA and proteins that promote tumour growth and provide diagnostic biomarkers. Nat. Cell Biol..

[152] Chalmin F., Ladoire S., Mignot G., Vincent J., Bruchard M., Remy-Martin J., Boireau W., Rouleau A., Simon B., Lanneau D. (2010). Membrane-associated Hsp72 from tumor-derived exosomes mediates STAT3-dependent immunosuppressive function of mouse and human myeloid-derived suppressor cells. J. Clin. Investig..

[153] Mathieu M., Martin-Jaular L., Lavieu G., Théry C. (2019). Specificities of secretion and uptake of exosomes and other extracellular vesicles for cell-to-cell communication. Nat. Cell Biol..

[154] Vickers K.C., Palmisano B.T., Shoucri B.M., Shamburek R.D., Remaley A.T. (2011). MicroRNAs are transported in plasma and delivered to recipient cells by high-density lipoproteins. Nat. Cell Biol..

[155] Zhuang X., Xiang X., Grizzle W., Sun D., Zhang S., Axtell R.C., Ju S., Mu J., Zhang L., Steinman L. (2011). Treatment of brain inflammatory diseases by delivering exosome encapsulated anti-inflammatory drugs from the nasal region to the brain. Mol. Ther..

[156] Liu C., Su C. (2019). Design strategies and application progress of therapeutic exosomes. Theranostics.

[157] Kooijmans S.A.A., Fliervoet L.A.L., van der Meel R., Fens M.H.A.M., Heijnen H.F.G., van Bergen En Henegouwen P.M.P., Vader P., Schiffelers R.M. (2016). PEGylated and targeted extracellular vesicles display enhanced cell specificity and circulation time. J. Control. Release.

[158] Guessous F., Alvarado-Velez M., Marcinkiewicz L., Zhang Y., Kim J., Heister S., Kefas B., Godlewski J., Schiff D., Purow B. (2013). Oncogenic effects of miR-10b in glioblastoma stem cells. J. Neurooncol..

[159] Nicoloso M.S., Spizzo R., Shimizu M., Rossi S., Calin G.A. (2009). MicroRNAs--the micro steering wheel of tumour metastases. Nat. Rev. Cancer.

[160] Ma L., Reinhardt F., Pan E., Soutschek J., Bhat B., Marcusson E.G., Teruya-Feldstein J., Bell G.W., Weinberg R.A. (2010). Therapeutic silencing of miR-10b inhibits metastasis in a mouse mammary tumor model. Nat. Biotechnol..

[161] Sheedy P., Medarova Z. (2018). The fundamental role of miR-10b in metastatic cancer. Am. J. Cancer Res..

[162] Callegari E., Elamin B.K., Giannone F., Milazzo M., Altavilla G., Fornari F., Giacomelli L., D’Abundo L., Ferracin M., Bassi C. (2012). Liver tumorigenicity promoted by microRNA-221 in a mouse transgenic model. Hepatology.

[163] Gallo Cantafio M.E., Nielsen B.S., Mignogna C., Arbitrio M., Botta C., Frandsen N.M., Rolfo C., Tagliaferri P., Tassone P., Di Martino M.T. (2016). Pharmacokinetics and Pharmacodynamics of a 13-mer LNA-inhibitor-miR-221 in Mice and Non-human Primates. Mol. Ther. Nucleic Acids.

[164] Bandi N., Zbinden S., Gugger M., Arnold M., Kocher V., Hasan L., Kappeler A., Brunner T., Vassella E. (2009). miR-15a and miR-16 are implicated in cell cycle regulation in a Rb-dependent manner and are frequently deleted or down-regulated in non-small cell lung cancer. Cancer Res..

[165] Reid G., Pel M.E., Kirschner M.B., Cheng Y.Y., Mugridge N., Weiss J., Williams M., Wright C., Edelman J.J., Vallely M.P. (2013). Restoring expression of miR-16: A novel approach to therapy for malignant pleural mesothelioma. Ann. Oncol..

[166] Ozcan G., Ozpolat B., Coleman R.L., Sood A.K., Lopez-Berestein G. (2015). Preclinical and clinical development of siRNA-based therapeutics. Adv. Drug Deliv. Rev..

[167] Cortez M.A., Ivan C., Valdecanas D., Wang X., Peltier H.J., Ye Y., Araujo L., Carbone D.P., Shilo K., Giri D.K. (2016). PDL1 Regulation by p53 via miR-34. J. Natl. Cancer Inst..

[168] Reid G., Kao S.C., Pavlakis N., Brahmbhatt H., MacDiarmid J., Clarke S., Boyer M., van Zandwijk N. (2016). Clinical development of TargomiRs, a miRNA mimic-based treatment for patients with recurrent thoracic cancer. Epigenomics.

[169] Winata P., Williams M., McGowan E., Nassif N., van Zandwijk N., Reid G. (2017). The analysis of novel microRNA mimic sequences in cancer cells reveals lack of specificity in stem-loop RT-qPCR-based microRNA detection. BMC Res. Notes.

[170] Rupaimoole R., Calin G.A., Lopez-Berestein G., Sood A.K. (2016). miRNA Deregulation in Cancer Cells and the Tumor Microenvironment. Cancer Discov..

